# Hope, despair and transformation: Climate change and the promotion of mental health and wellbeing

**DOI:** 10.1186/1752-4458-2-13

**Published:** 2008-09-17

**Authors:** Jessica G Fritze, Grant A Blashki, Susie Burke, John Wiseman

**Affiliations:** 1 Department of General Practice, University of Melbourne, Victoria 3010 Australia; 2 Visiting Fellow at National Centre for Epidemiology and Population Health, The Australian National University, Canberra, ACT, 0200, Australia; 3 Australian Psychological Society, Melbourne, Victoria, Australia; 4 Research Fellow, McCaughey Centre: VicHealth Centre for the Promotion of Mental Health and Community Wellbeing, University of Melbourne, Victoria, Australia; 5 Director, McCaughey Centre: VicHealth Centre for the Promotion of Mental Health and Community Wellbeing, University of Melbourne, Victoria, Australia

## Abstract

**Background:**

This article aims to provide an introduction to emerging evidence and debate about the relationship between climate change and mental health.

**Discussion and Conclusion:**

The authors argue that:

i) the direct impacts of climate change such as extreme weather events will have significant mental health implications;

ii) climate change is already impacting on the social, economic and environmental determinants of mental health with the most severe consequences being felt by disadvantaged communities and populations;

iii) understanding the full extent of the long term social and environmental challenges posed by climate change has the potential to create emotional distress and anxiety; and

iv) understanding the psycho-social implications of climate change is also an important starting point for informed action to prevent dangerous climate change at individual, community and societal levels.

## Background

While the burden of disease of mental illness has long been recognised, there is an increasing understanding of the social, economic and environmental determinants that promote the mental health of individuals, communities and societies [1-3]. As evidence about the reality and the consequences of climate change has expanded, a growing body of evidence has emerged about the health effects of climate change [4]. In this article we focus particularly on the *mental health *implications of climate change.

This article will therefore discuss three areas of emerging concern about the relationship between climate change and mental health. Firstly, the direct impacts of climate change, such as extreme weather events, are likely to have immediate impacts on mental health concerns and outcomes. Secondly, vulnerable communities are beginning to experience disruptions to the social, economic and environmental determinants that promote mental health. Finally, there is an emerging understanding of the ways in which climate change as a global environmental threat may create emotional distress and anxiety about the future. We conclude with some reflections on future policy, practice and research priorities in the fields of mental health and mental health promotion.

### Health and climate change

There is now a very strong scientific consensus that global warming is occurring, that it is largely caused by human emissions of greenhouse gases, that the effects are already observable, and that further warming will occur. The most recent report of the Intergovernmental Panel on Climate Change (IPCC) estimates current global warming to be almost 0.8°C above pre-industrial levels and projects a further rise in the coming decades [5]. This report also reconfirms that it is human emission of greenhouse gases that has been mostly responsible for global warming over the past 50 years, and that even if emissions are greatly curtailed, the existing accumulation of emissions in the Earth's atmosphere has committed us to some warming over the coming century [5].

The impacts of current global warming are now observable in physical systems such as the rise of sea levels, glacial retreat, significant reductions in the extent and thickness of Arctic sea ice, alterations in rainfall patterns and in biological systems such as earlier spring activities of numerous plant and animal species [5]. There is growing concern that the current growth rate of greenhouse gas emissions and observed climate impacts indicate that climate change is occurring at a rate greater than the most pessimistic scenarios assessed by the IPCC [6,7].

Research on the health effects of climate change has focused largely on direct physical health impacts, principally:

• death and injury from extreme weather events;

• impacts of increased temperatures and heat waves;

• spread of vector-borne disease;

• air quality and respiratory illness; and

• changes in food and water quality and availability [4,8].

There is also increasingly clear recognition that the distribution of health impacts will tend to fall more heavily on low-income or otherwise more vulnerable populations [8,9].

### Mental health and climate change: short and long term implications

The psychological and mental health implications of climate change have only more recently been considered within climate change and health frameworks, particularly in the context of disaster recovery from extreme weather events [8,10]. In the Australian context, the broader connections between climate change and mental health have also importantly begun to be canvassed as part of the Garnaut Climate Change Review [11].

There are three key mental health implications of climate change. Firstly, direct impacts of climate change, such as extreme weather events, are likely to have immediate impacts on the prevalence and severity of mental health issues in affected communities as well as significant implications for mental health systems. Secondly, vulnerable communities are beginning to experience disruptions to the social, economic and environmental determinants that promote mental health. Finally, there is an emerging understanding of the ways in which climate change as a global environmental threat may create emotional distress and anxiety about the future.

## Discussion

### Immediate mental health impacts of climate change

There is an extensive body of evidence showing the ways in which extreme weather events can lead to psychological and mental health outcomes associated with loss, disruption and displacement as well as cumulative mental health impacts from repeated exposure to natural disasters [10,12-16]. Disaster response and emergency management have been a focus of government and agencies over the past decade, with an increasing emphasis on psychological and psychosocial interventions [17].

Mental health impacts differ according to the type, suddenness and scale of the catastrophe, and the social, historical and cultural context in which it occurs [18]. Impacts are compounded by the vulnerability of individuals and communities, the appropriateness of emergency responses, and the resources available to provide support and rebuild. While extreme weather events occur world wide, it is often the poorest communities that are already deficient in services, which are likely to be disproportionately impacted by natural disasters.

Despite cultural variations between countries and individuals, communities show some common patterns of psychosocial responses to disasters [13]. Acute traumatic stress is the most common normative response post disaster, with symptoms subsiding once conditions of safety and security have been re-established [19]. Some survivors, however, will continue to experience chronic post-traumatic stress disorder (PTSD), as well as a range of other stress-related problems such as complicated grief, depression, anxiety disorders, somatoform disorders and drug and alcohol abuse [20]. Children often exhibit more severe distress after disasters than do adults [21].

A preliminary overview of research conducted in communities effected by Hurricane Katrina shows high rates of depression, domestic violence and significantly higher rates of suicide completion and attempts (14.7 and 78.6 times the area's baseline rate respectively) [22]. This population also showed high rates of post-traumatic stress disorder [23]. These mental health outcomes are attributable not simply to exposure to the event, but subsequent displacement, unstable housing, and lack of access to support services and employment [22]. A study of child abuse following North Carolina's Hurricane Floyd concluded that families are vulnerable to an elevated risk of child abuse following a disaster [24], probably related to increased parental stress and decreased social support.

Caring for the mental health needs of affected people following an extreme weather event, or other natural disaster, is constrained by several factors, such as limitations in service capacity (resources and skills), high levels of immediate chaos, widespread distress, and delays before essential services are re-established [20]. Emergency mental health services need to combine several interventions to meet the needs of a distressed community, as well as the needs of those who are traumatised and those with severe mental illness [17]. There is ongoing debate about where and how services should be prioritised [25]. Consensus is emerging about the importance of rapidly restoring safety and security so that the majority of the population can begin the recovery process. This effectively reduces the numbers of people with traumatic stress reactions. The ADAPT model (Adaptation and Development after Persecution and Trauma) proposes that the ideal recovery environment is one which repairs the key psychosocial domains that are threatened by disasters. This includes creating conditions of security and safety, reuniting families, establishing systems of justice, creating foundations for work/livelihoods, and restoring institutions that confer existential meaning and coherence [26]. For the majority of people, this type of care is sufficient.

People with pre-existing severe mental health issues, such as psychosis, are especially vulnerable to significant mental health impacts following a disaster. These people need urgent care and can present a significant challenge in an emergency context where pre-existing mental health services may have been destroyed and severely disrupted by the disaster.

More recently, interest in mental health interventions post disaster has also included pre-event community education and preparedness [17]. Significant mental health issues are experienced by members of communities in disaster prone areas, particularly during 'disaster seasons' where people have to live with ongoing uncertainty, anxiety and dread before a disaster has even occurred. These significant background stressors are routinely underestimated or ignored in the literature [27]. There is an urgent need for better understanding of the mental health impact of ongoing climate-change 'threats' on individuals and communities living in disaster prone regions.

### Impact of climate change on social and economic determinants of mental health

Increasing awareness of the importance of understanding and addressing mental health promotion prompts us to think more widely about the impact of climate change on the broader determinants of mental health.

The World Health Organisation (WHO) defines health as 'a state of complete physical, mental and social well-being and not merely the absence of disease or infirmity' [3]. The 1986 Ottawa Charter for Health Promotion builds on this definition in the following way:

*To reach a state of complete physical, mental and social wellbeing, an individual or group must be able to identify and to realise aspiration, to satisfy needs, and to change or cope with the environment. Health is, therefore, seen as a resource for everyday life, not the objective of living. Health is a positive concept emphasising social and personal resources, as well as physical capacities. Therefore, health promotion is not just the responsibility of the health sector, but goes beyond healthy lifestyles to wellbeing *[28].

The *Mental Health Promotion Framework *developed by the Victorian Health Promotion Foundation (VicHealth) applies this understanding to the field of mental health, noting that:

*Mental health is not merely the absence of mental illness. Mental health is the embodiment of social, emotional and spiritual wellbeing. Mental health provides individuals with the vitality necessary for active living, to achieve goals and to interact with one another in ways that are respectful and just *[29].

Informed by an extensive review of available evidence, VicHealth has identified a number of potentially modifiable social and economic determinants crucial to the mental health and wellbeing of individuals and communities: economic security and participation, social inclusion and freedom from violence and discrimination [29]. The following discussion outlines potential impacts of climate change and of climate adaptation and mitigation policies on these broad determinants of mental health and wellbeing.

#### Economic security and participation

*While mainstream debate currently focuses on science and the economic dimensions of climate change there is also growing awareness of its social costs. There is mounting understanding that the effects of climate change will be disproportionately felt by already vulnerable communities, including people on low incomes and communities directly dependent on their local environment for survival *[9].

Relative socioeconomic disadvantage and unemployment are linked to poor mental health through increased exposure to psychosocial risk factors including reduced personal autonomy, negative self perception, stress, insecurity and social isolation [30-32]. Through its likely impact on economic systems and cost of living, and the unequal distribution of these impacts within and between communities, climate change is likely to negatively impact on mental health and wellbeing.

Financial hardship related to climate change may result from: reduced income and employment in climate sensitive industries such as agriculture and tourism; loss of assets and recovery costs from extreme weather events or relocation; and increases to the cost of essential goods and services [9,33-35]. In addition, the rapid restructuring of emissions intensive industries including power generation, agriculture and heavy industry present significant challenges for mental health through potential loss of employment in these industries, especially in low-skilled or regional workforces with few employment alternatives.

While some communities are more likely to be exposed to climate change impacts due to their location (for example, coastal areas), others have limited adaptive capacity due to poverty, poor physical and service infrastructure and economic reliance on climate vulnerable ecosystems [36]. Some communities are vulnerable on both counts, and it is in these communities that the social and economic impacts of climate change are likely to be most severe. Communities already experiencing socioeconomic disadvantage and associated higher rates of mental health issues are therefore likely to be further marginalised by economic impacts of climate change, which in turn has implications for community wellbeing and mental health systems.

Reduced income security caused by climate change is likely to be exacerbated by increased costs of basic goods and services, leading to greater economic exclusion. Climate change can affect food security through disruptions to the global food supply from extreme weather events but also through relatively rapid changes to the viability of agricultural land. Recent rapid increases in food prices due to drought and global demands for biofuels have highlighted the vulnerability of the world's food production systems to climate change [37].

Other basic goods and services such as energy, water and communications are likely to become less affordable as production and distribution costs rise and competition increases for water resources [38]. Insurance costs are also likely to rise due to increased risk within the insurance industry. These rises may further expose low income households, many of whom are already under-insured, to mental health impacts from the irrecoverable loss of possessions as well as significant financial stress [39].

The interaction of climate related economic impacts and mental health is already being felt in drought effected rural Australia. Reduced income security due to ongoing drought has contributed to a number of social impacts including stress, social isolation, strain on relationships, and evidence of increased rates of suicide [40-42]. Farm families are employing a number of economic coping strategies, such as seeking work outside the agricultural sector and reducing household expenditure. However, without adequate support, these strategies in themselves may have mental health impacts. Reducing participation in 'optional' activities such as social events can contribute to isolation, while increased workloads and separation of families to access employment opportunities creates additional emotional stress [43].

Inadequate service and policy responses to support communities economically affected by climate change and necessary economic restructuring are likely to increase the negative impacts on wellbeing at both an individual and community level.

#### Social exclusion and effects of displacement

Social exclusion refers to a multidimensional lack of connection with the activities of the wider community and encompasses lack of economic participation, social disconnection and lack of access to services [44]. In addition to the effects on economic participation outlined above, climate change is likely to fracture social networks and community connection through the increased displacement of climate vulnerable communities as a result of both economic and forced migration.

Quantifying involuntary migration due to climate change is extremely difficult as many existing factors such as social and economic vulnerability, security of food and water supplies, and capacity to migrate are likely to interact with physical climate change impacts to influence rates and types of displacement [45]. However, there is little doubt that both the long-term effects of climate change and associated extreme weather events will displace significant numbers of people, largely from already vulnerable communities. Extreme weather events, sea level rise, destruction of local economies, resource scarcity and associated conflict due to climate change are predicted to displace millions of people worldwide over the coming century. The most commonly cited figure of projected population displacement from climate change is 200 million people by 2050 [45,46].

The negative mental health impacts of forced migration, whether fleeing from violence, destroyed livelihoods or extreme poverty are significant though vary greatly depending on individual circumstances [47]. The loss of connection to place and sense of belonging associated with displacement can also undermine mental health [48].

*Unlike survivors of most discrete traumatic events, refugees experience diverse stressors that accumulate over the preflight, flight, exile, and resettlement/repatriation periods. Despite the historical focus on the acute stressors of war, the enduring contextual postmigration stress that refugees face – including marginalization, socioeconomic disadvantage, acculturation difficulties, loss of social support, and "cultural bereavement" – must be recognized *[49].

This has implications for refugee and resettlement policy, in particular the resources available to support communities following migration. Porter and Haslam (2005) note that availability of support and economic opportunities in receiving countries are linked to better mental health outcomes for newly arrived communities [49].

Increased exposure to cultural and racial discrimination in receiving communities is also a risk factor for negative mental health outcomes. This is especially likely if immigrants are seen as a cultural threat or competition for natural or economic resources, as may well be the case in climate change situations [46].

Within Australia, the internal displacement of some climate vulnerable communities is increasingly likely, especially from rural communities, coastal areas and the Torres Strait. The loss of population and community vitality associated with economic migration from small rural communities is likely to affect the mental health of both those displaced in search of more stable livelihoods and those remaining in the declining community [50]. The economic and health effects of climate change are likely to be compounded for remote Indigenous communities, many of which currently lack sufficient social and physical infrastructure and experience profound socioeconomic disadvantage. For Indigenous communities living in climate vulnerable coastal areas, climate change may have additional mental health impacts associated with disruption to traditional practices, damage to significant cultural sites and, in the long-term, potential relocation [51].

*In the long term, there are also significant cultural impacts caused by Indigenous communities' inability to feel able to maintain the health of their land and sea country. The psychological effect of such loss on individuals and families is likely to cause a heavy burden of distress and mental illness in many communities *[52].

#### Exposure to violence

Psychological distress, loss and displacement resulting from violent conflict are unsurprisingly associated with a wide range of mental health impacts including anxiety, depression and PTSD [53].

At this point, there are conflicting views on whether a direct causal relationship exists between climate change and violent conflict [54]. However, climate change is likely to undermine human security by 'reducing access to natural resources that are essential to sustain... livelihoods' [55]. Increased competition for scarce resources, especially water, and forced migration have historic links to increased violent conflict [53,56]. Stern (2006) notes that increased climate variability as well as overall climate deterioration could contribute to increased violence:

*the effects of climate change – particularly when coupled with rapid population growth, and existing economic, political, ethnic or religious tensions – could be a contributory factor in both national and cross-border conflicts in some developing countries *[34].

### Emotional distress arising from awareness of climate change as global environmental threat

The longer term impact of climate change on mental health comes from people's emerging *awareness *of climate change as a global environmental threat – not the experience of climate change events per se. As people's understanding of climate change grows and deepens, it is likely to have a significant impact on their social, emotional and spiritual wellbeing.

Bill McKibben's reflections on the 'end of nature' provide an important starting point for understanding this challenge.

*Our comforting sense of the permanence of our natural world, our confidence that it will change gradually and imperceptibly if at all, is the result of a subtly warped perspective. Changes that can affect us can happen in our lifetime in our world-not just changes like wars but bigger and more sweeping events. I believe that without recognizing it we have already stepped over the threshold of such a change; that we are at the end of nature. By the end of nature I do not mean the end of the world. The rain will still fall and the sun shine, though differently than before. When I say 'nature,' I mean a certain set of human ideas about the world and our place in it *[57].

The question that McKibben raises is how psychologically, emotionally and politically should we as human beings respond to this fundamental change in the relationship between the human species and the world we inhabit?

It is also important to note that popular understanding of climate change is primarily mediated through communication channels (television, computer, radio, newspapers and magazines), which filter the way the public views climate change. The ways in which these stories are framed, edited and represented, impact on how an individual understands climate change as well as the ways in which they respond to this news.

Individuals further make sense of the information that they receive about climate change by talking with others, sharing views, and reality testing their appraisals. In the end, an individual's understanding of climate change, and their emotional reactions to this knowledge, is a combination of individual processes such as their own concerns, defenses, thoughts and feelings, and social processes. Some of these social processes include social constructions of climate change and social amplification of risk in which public perceptions of risk are intensified or dampened [27].

There is a complex relationship, therefore, between climate change and people's awareness of, and responses to, environmental threats. From a psychological point of view, feelings and thoughts about such a potentially enormous threat are likely to be 'managed' by adaptive protection motivation systems, and modified through social comparison with others and selective information exposure [27].

For many people, the resulting emotions are commonly distress and anxiety. People may feel scared, sad, depressed, numb, helpless and hopeless, frustrated or angry. Sometimes, if the information is too unsettling, and the solutions seem too difficult, people can cope by minimising or denying that there is a problem, or avoiding thinking about the problems. They may become desensitised, resigned, cynical, skeptical or fed up with the topic. The caution expressed by climate change skeptics could be a form of denial, where it involves minimising the weight of scientific evidence/consensus on the subject. Alternatively, it could indicate that they perceive the risks of change to be greater than the risks of not changing, for themselves or their interests [58].

Spratt and Sutton usefully extend this discussion of the political implications of 'climate change denial' further, noting:

*all these forms of blocking lead to a society accepting inaction or insufficient action as a solution to the problem. The alternative – to imagine and plan for a great transformation of our society in a way that is consistent with safe climate future – may be unsettling and challenging. It will require us to change the way we live and how we understand the relationship between our actions and out future on this fragile planet. But it is the only practical option*. [7]

The psychological impact of climate change as a global phenomenon has a lifecycle aspect. Children and young people growing up with an uncertain future that is not of their making may experience the threat of climate change very differently from their parents and grandparents. As Tucci, Mitchell and Goddard (2007) note in a survey of Australian children 'a quarter of children are so troubled about the state of the world that they honestly believe it will come to an end before they get older' [59].

It is likely that many children are aware of the threat of climate change. However, it is also quite likely that they are confused about the facts and the magnitude of the threat they personally face, and might feel anxious, concerned or confused. Worries and anxieties about these threats can become difficult for children of all ages to deal with.

*We can measure and communicate the effects of the threat of global environmental destruction on the current mental health of our children. At the height of the Cold War, when nuclear war appeared imminent, through accident or pre-emptive strike, school children reacted with despair and loss of motivation. Many children thought they would not survive to adulthood. We can actively contribute to the debate on global warming by providing good data on this specific issue, but the nuclear weapons experience suggests that our children and grandchildren will react to expanding knowledge of climate change with despair *[60].

The comparison between the emotional and psychological impacts of the threat of nuclear war and climate change is valuable and revealing. In both instances there is a real and imminent threat not just to the survival of particular individuals, communities or societies but to the human species – and perhaps to life on the planet as a whole.

However, a significant difference between the threats of nuclear war and climate change is the potentially greater range of ways in which citizens have the capacity to take action individually and collectively. Notwithstanding the enormity of the climate change challenge, we know what many of the solutions are, and there are many actions that citizens can take individually and collectively to make a difference at household, local, national and global level. When people have something to do to solve a problem, they are better able to move from despair and hopelessness to a sense of empowerment.

### Mental health and climate change: policy, practice and research implications and challenges

Emerging evidence of the mental health and psycho-social implications of climate change can usefully inform understanding and action at individual, community and societal levels. The following concluding remarks provide an initial sketch of potential directions and issues requiring further research and debate.

At the level of individual responses, the Australian Psychological Society has developed the following advice and suggestions for mental health professionals and individuals experiencing psychological and emotional distress in relation to the threat of climate change (see Table 1). These tips are derived from an extensive psychological literature including social and behavioural change literature, environmental and community psychology, and the positive psychology movement. The tips are not necessarily useful or appropriate for individuals with mental health issues, or who are experiencing severe levels of anxiety or depression, for whom professional help would be more appropriate.

**Table 1:** Climate change: What you can do. Australian Psychological Society

'Although environmental threats are real and can be frightening, remaining in a state of heightened distress is not helpful for ourselves or for others. We generally cope better, and are more effective at making changes, when we are calm and rational.'

• Be optimistic about the future
• Remind yourself there is a lot you can personally do
• Change your own behaviour
• Become informed about problems and solutions
• Do things in easy stages
• Identify things that might get in the way of doing things differently
• Cue yourself
• Look after yourself!
• Invite others to change
• Talk with others about environmental problems
• Present clear but not overwhelming information, and offer solutions
• Talk about changes that you are making in your own life
• Share your difficulties and rewards
• Be assertive, not aggressive
• Congratulate people for being environmentally concerned
• Model the behaviours that you want others do do' [58]

From a clinical perspective, an emerging area of research is management of individuals with excessive worry or anxiety about climate change. Whilst a heightened level of concern in the general community is not unexpected and indeed appropriate, some individuals experience more intense worry that causes distress and/or interferes with normal day to day life. Anecdotally, groups that appear to be more at risk include individuals with existing depression or anxiety disorders, those working in the field of climate change, and children and adolescents.

Therapeutic approaches will vary greatly depending on the clinical presentation, the background and training of health professional, their affiliation to a particular psychological orientation and the clinical setting in which the individual presents. For example, psychological approaches may be theoretically grounded in eco-psychology, grief/loss counselling, cognitive behavioral therapy, interpersonal therapies, group therapy approaches aimed at building social capital and resilience, or a range of other approaches. Overlaying this diversity of theoretical approaches is the therapist's own conceptualisation of environmental issues, which is likely to affect how they see their role and to some extent, the aims of the therapy.

As noted above the capacity of mental health systems to respond effectively to the short and longer term impacts of climate change will be influenced by a number of factors including the availability of an appropriately skilled workforce and the extent to which services are linked and integrated with other relevant services and organisations [35]. This will be a challenging policy area in Australia given that mental health services are already under resourced to meet existing demand, especially in rural areas where recruitment and retention of mental health professionals is particularly difficult [35,61].

In thinking about opportunities for community and societal level actions, many governments, communities and environmental organisations have begun to develop integrated strategies for climate mitigation and adaptation informed by a commitment to creating healthy, just and sustainable outcomes. The Southern Grampians and Glenelg Primary Care Partnership based in rural south-west Victoria has, for example, developed a strategic framework for local community climate change adaptation [62]. The Westernport and North East Greenhouse Alliances have undertaken similar local vulnerability and adaptation planning, informed by input from a wide range of local community, private and public sector organisations [63,64].

Resources to guide and inform similar local adaptive planning initiatives will be important, including:

• advice on the best ways of engaging individuals, communities and organisations in developing well informed responses and strategies;

• up to date, accessible information on local climate change impacts, scenarios and tipping points; and

• mechanisms for sharing learning about the most effective local and regional level actions.

While local level action to reduce socioeconomic vulnerability, physical and mental health impacts is essential for effective climate change adaptation, actions at the local level are often constrained by funding and policy environments determined by higher levels of government.

The climate change adaption strategy developed by the Western Australian Department of Health [35] is an example of the kind of state level policy response needed to ensure that social and mental health impacts of climate change are adequately addressed. Specific policy recommendations in this strategy are included in the areas of:

• legislation and regulatory reform;

• public education and communication;

• surveillance and monitoring;

• ecosystem intervention;

• infrastructure development; and

• health intervention.

Other areas of social and economic policy require similar attention. The best options to support individuals, workers, families and communities through economic structural adjustment and potential migration necessitated by climate change and climate policy, especially in drought effected or fossil fuel industry based communities, need to be established and implemented early to minimise economic and social exclusion.

Given the variation of climate change impacts across different areas and population, effective policy making and resource allocation to reduce mental health impacts will require a solid research base. Key research questions in relation to the impact of climate change on mental health include the following.

• What are the key mental health and mental health promotion impacts and implications of the most probable climate change scenarios? What are the implications of climate change for the key social and economic determinants of mental health and community wellbeing?

• What are the implications for particular localities and for the most vulnerable and excluded population groups?

• What are the key mental health and mental health promotion impacts of proposed climate change mitigation and adaptation strategies?

Issues of design and methodology will also be important considerations in developing an effective research agenda for understanding the social justice implications of climate change. How, for example, should participatory and collaborative research strategies be employed in the design and implementation of research projects? What kinds of indicators, data sets and data analysis techniques will be needed to predict and monitor the health and mental impacts of climate change? How can the results of research be most effectively communicated and translated in ways that rapidly and effectively inform public debate and policy choices? Interdisciplinary approaches to assessing risk, vulnerability and resilience of communities exposed to climate change impacts will also enhance policy development and planning.

### Climate change, hope, despair and transformation

As Eckersley notes:

*there is a real and increasing possibility that climate change, resource depletion, increasing world population, disease pandemics, technological anarchy and the geopolitical tensions, economic instability and social upheaval they generate will create a nightmare future for humanity in this century. Avoiding this fate will depend critically on the stories we create to make sense of what is happening and to frame our response. A key task is to ensure that these stories reflect neither the decadence and degeneracy of nihilism nor the dogma of fundamentalism but the hope and creative energy of activism *[65].

In the long-term, hope and morale in the community about climate change is deeply intertwined with mental health promotion. For the community to be less pessimistic about the future requires a realistic understanding of what climate change means and what can be done. At a time when the predictions from our most credible scientists are becoming increasingly grave, those involved in mental health promotion need to pay close attention to the relation between evidence, hope and action.

At the deepest level, the debate about the consequences of climate change gives rise to profound questions about the long-term sustainability of human life and the Earth's environment. If our view about the future of human society and the planet is fundamentally pessimistic what does this mean for the way in which a sense of hope – or despair – for future generations impacts on the sense of individual and collective meaning and purpose?

On the other hand, the challenges of climate change adaptation may galvanise creative ideas and actions in ways that transform and strengthen the resilience and creativity of individuals and communities. Seligman's work [66] on positive psychology, with its focus on the relationship between mental health, hope and optimism, provides strong support for the potential for growth and transformation to emerge from the climate crisis. As a common threat, climate change may provide an impetus for collaborative action within communities, and indeed internationally. Climate change may unite communities in action against a common threat, or create social and political instability in competition for increasingly scarce environmental resources.

A broader debate is required about the relationship between ecological, economic and social justice principles and objectives. As Richard Eckersley (2008), Clive Hamilton, (2006), George Monbiot (2007) and David Spratt and Phillip Sutton (2008) have all noted, a full understanding of the consequences and implications of climate change calls for a response that extends well beyond technical fixes and cost benefit analyses [65,67,7,68]. The larger – and increasingly urgent – challenge is to begin to envisage and build social and economic relationships that are based on sustainable and just patterns of growth and consumption.

## Competing interests

The authors declare that they have no competing interests.

## Authors' contributions

GB and JW conceived the paper and developed a first draft. JW coordinated the development process. JF undertook the literature review, developed the paper and incorporated co-author's contributions. SB contributed generally to successive iterations of the paper and specifically to table 1. All authors read and approved the final manuscript.
